# Recombinant Swinepox Virus Expressing Glycoprotein E2 of Classical Swine Fever Virus Confers Complete Protection in Pigs upon Viral Challenge

**DOI:** 10.3389/fvets.2017.00081

**Published:** 2017-05-30

**Authors:** Huixing Lin, Zhe Ma, Lei Chen, Hongjie Fan

**Affiliations:** ^1^College of Veterinary Medicine, Nanjing Agricultural University, Nanjing, China; ^2^Jiangsu Co-Innovation Center for Prevention and Control of Important Animal Infectious Diseases and Zoonoses, Yangzhou, China

**Keywords:** classical swine fever virus, swinepox virus, glycoprotein E2, vaccine, pigs

## Abstract

Classical swine fever (CSF) is a highly contagious and serious viral disease that affects the pig industry worldwide. The glycoprotein E2 of the classical swine fever virus (CSFV) can induce neutralizing antibodies, and it is widely used for novel vaccine development. To explore the development of a vaccine against CSFV infections, the gene of glycoprotein E2 was inserted into the swinepox virus (SPV) genome by homologous recombination. The culture titers of rSPV-E2 remained at about 4.3 × 10^6^ TCID_50_ for more than 60 passages in PK15 and swine testis cell lines. The rSPV-E2 could not be replicated in Vero, MDBK or other non-porcine cell lines. After two to three passages, the SPV specific gene of rSPV-E2 could not been detected in the non-porcine cell culture. To evaluate the immunogenicity of rSPV-E2, 20 CSFV seronegative minipigs were immunized with rSPV-E2, a commercial C-strain vaccine, wild-type SPV (wtSPV; negative control), or PBS (a no-challenge control). After challenge with CSFV, pigs in the rSPV-E2-immunized group showed significantly shorter fever duration compared with the wtSPV-treated group (*P* < 0.05). E2-specific antibodies in the rSPV-E2-immunized group increased dramatically after vaccination and increased continuously over time. CSFV genomic copies in the serum of rSPV-E2-immunized pigs were significantly less compared with the wtSPV-treated group at all time points after challenge (*P* < 0.01). Significant reduction in gross lung lesion scores, histopathological liver, spleen, lung, and kidney lesion scores were noted in the rSPV-E2-immunized group compared with the wtSPV-treated group (*P* < 0.01). The results suggested that the recombinant rSPV-E2 provided pigs with significant protection from CSFV infections; thus, rSPV-E2 offers proof of principle for the development of a vaccine for the prevention of CSFV infections in pigs.

## Introduction

Classical swine fever (CSF) is one of the most serious infectious diseases of domestic pigs worldwide, and it is characterized by highly contagious, multisystem hemorrhage and immunosuppression (1, 2). Vaccination with live attenuated vaccines, such as the C-strain, protects pigs from clinical CSF disease (3, 4). The current strategies to control CSF are prophylactic vaccination and the stamping-out strategy without prophylactic vaccination (5). The Hog cholera lapinized virus (HCLV) vaccine, also known as the Chinese vaccine strain (C-strain), is a modified live vaccine. HCLV was attenuated from a highly virulent strain (disputably Shimen strain) after at least 480 passages in rabbits (6). Because of its high efficacy and safety, the HCLV vaccine has been widely used to prevent CSF in many of the endemic countries including China. However, use of the HCLV vaccine does not allow discrimination of vaccinated and infected animals. Therefore, generation of a vaccine that enables differentiation of infected from vaccinated animals (DIVA) would benefit CSF control and eradication program, particularly in the later stages of an eradication campaign and for countries where the disease is not endemic.

Classical swine fever virus (CSFV) is a member of the genus *Pestivirus* within the family *Flaviviridae* (7). The glycoprotein E2 of CSFV is essential for viral replication and infection (8, 9), and it is also the major immunogenic protein for inducing neutralizing antibodies to elicit protective immunity against CSFV (10, 11). Previous studies have shown that the E2 envelope protein contains conserved epitopes that induce CSFV-neutralizing antibodies (12–14).

Swinepox virus (SPV) possesses a 146 kb double-stranded DNA genome, and it replicates in the cytoplasm of the host cell (15). SPV infects only swine, and natural SPV infections are typically mild; they are occasionally accompanied by localized skin lesions, which heal naturally. SPV is well suited for the development of recombinant vaccines due to its large packaging capacity for recombinant DNA and its ability to induce appropriate immune responses (16, 17). In this study, a recombinant swinepox virus expressing glycoprotein E2 (rSPV-E2) of CSFV was constructed, and the potential of using the recombinant SPV as a porcine vaccine candidate against CSFV infections was explored.

## Materials and Methods

### Cells and Viruses

Swinepox virus (VR-363) was purchased from the American Type Culture Collection (ATCC). The highly virulent CSFV Shimen strain was obtained from the Control Institute of Veterinary Bioproducts and Pharmaceuticals (Beijing, China). Porcine kidney cells (PK15), swine testis cells (ST), mouse lung cancer cells (LLC), baby hamster kidney cells (BHK-21), Chinese hamster ovary cells (CHO), African green monkey kidney cells (Vero), human laryngeal epidermoid carcinoma cells (HEp-2), human cervical cancer cells (HeLa), Madin–Darby canine kidney cells (MDCK), rabbit kidney cells (RK13), bovine kidney cells (MDBK), chicken embryo fibroblast cells (CEF), and feline kidney cells (F81) were purchased from the ATCC or the Cell Bank of the Chinese Academy of Sciences (Shanghai, China), respectively. The cells were routinely cultured at 37°C in 5% CO_2_ in Eagle’s Minimum Essential Medium supplemented with 5–10% fetal bovine serum (FBS, Gibco).

### Animals and Housing

Bama minipigs were widely used in vaccine evaluation and pathogenicity study (18, 19). Twenty clean-grade Bama minipigs (4-week-old) were purchased from the Shanghai Academy of Agricultural Sciences. They were randomly divided into four groups and housed in four separate rooms. All experimental protocols were approved by the Laboratory Animal Monitoring Committee of Jiangsu province and performed accordingly (Government Degree No. SYXK2015-0036).

### Construction and Identification of rSPV-E2

RNA was extracted from the CSFV C-strain vaccine (TechBank Biotech, Nanjing, China). A pair of primers, E2F: *GTCGAC*GCCACCATGGCATCAACCATTGCATTCCT (containing *Sal*I site and kozak sequence) and E2R: *GGATCC*TTATTAACCAGCGGCGAGTTGTT (containing stop codon and *Bam*HI site), were used for CSFV E2 gene amplification. The 1,218 bp RT-PCR products were cloned into the *Sal*I/*Bam*HI sites of the pUSG11/P28 vector (19), generating the recombinant plasmid pUSG11/P28-E2. The recombinant SPV, rSPV-E2, was constructed by homologous recombination of wild-type SPV with pUSG11/P28-E2 as previously described (20). The expression of glycoprotein E2 was analyzed by Western blot and indirect immunofluorescence as previously described (20). Briefly, PK15 cells grown on a 12-well plate were infected with the wtSPV or recombinant virus rSPV-E2 (15 PFU per well). At 72 h postinfection, cells were washed twice in PBS and fixed with cold methanol for 10 min at −20°C. Cells were then washed three times with PBST and blocked by the addition of 10% BSA in PBST. Preparations were incubated for 1 h at 37°C with the monoclonal antibody against E2 (Abnova) in dilution buffer (1% BSA in PBST). After three washes with PBST, cells were treated with the rhodamine conjugated secondary antibody (goat anti-mouse IgG-R, Cwbio) 1:5,000 dilution with PBS for 30 min at 37°C. After a final wash with PBS, all wells were examined using fluorescence microscopy (Zeiss, Germany).

### *In Vitro* Analyses of Proliferative Capacity and Genetic Stability of rSPV-E2

PK15, Vero and the other cells in 25 cm^2^ cell culture flasks were infected with 0.01 multiplicity of infection of rSPV-E2. The cells were incubated at 37°C for 2 h. The infection medium was discarded and 10 mL DMEM medium containing 2% FBS was added to the cell culture. When 50% of the PK15 cells showed cytopathic lesions (about 72 h), the cells were freeze-thawed twice. The cell culture medium containing rSPV-E2 was collected and used to inoculate another uninfected PK15 monolayer; this way, rSPV-E2 was cultured for 60 passages. The titer of SPV rSPV-E2 in cell culture medium for each passage was determined, and the whole genome sequencing was carried out every 10 passages to evaluate its genetic stability.

### Animal Experiment

The piglets were randomly divided into four groups using a random numbers generated by SPSS 19.0 and housed in four separate rooms. Group 1 (*n* = 5) was vaccinated intramuscularly with 2 mL of rSPV-E2 by 2 × 10^6^ TCID_50_ per piglet. Group 2 (*n* = 5) was vaccinated intramuscularly with 2 mL of the commercial C-strain vaccine per piglet (TechBank Biotech, Nanjing, China). Group 3 (*n* = 5) was vaccinated intramuscularly with 2 mL of wtSPV by 2 × 10^6^ TCID_50_ per piglet. Group 4 (*n* = 5) was treated with 2 mL of PBS as the no-challenge control (NC). The piglets were boosted once at 14 days after the first vaccination following the same immunization protocols. After vaccination, sera samples were collected at 7-day intervals for detection of E2-specific antibodies and neutralizing antibodies against CSFV. At 0, 14, 28, and 35 days post-immunization (dpi), blood was collected from each piglet, and peripheral blood mononuclear cells (PBMCs) were separated for cellular immune response detection. At 35 days post-primary immunization, groups 1, 2, and 3 were intranasally challenged with 1 × 10^5^ TCID_50_ CSFV Shimen strain per piglet. The rectal body temperature and clinical signs were monitored daily. The serum samples were collected at 0, 4, 7, 10, and 14 days post-challenge (dpc) for viremia analysis. The piglets showing signs of irreversible illnesses, such as pyrexia (>40°C), weakness, anorexia, systemic congestion, and convulsions, were humanely euthanized with 100% concentration of CO_2_, and then underwent pathological and histopathological examination following standard operational procedures.

### Humoral Immune Response Detection

#### Blocking ELISA

Classical swine fever virus-specific antibodies present in pig serum samples were tested using a CSFV antibody ELISA kit (Blocking ELISA based on E2 MAb, IDEXX) according to the manufacturer’s instructions.

#### Virus Neutralizing Antibody Detection

Sera samples were collected at 7-day intervals postimmunization and heat-inactivated for 30 min at 56°C. Replicates of twofold serially diluted sera (starting from 1/4) were mixed with an equal volume of 100 TCID_50_ of CSFV Shimen strain and incubated at 37°C for 1 h. Each of the mixtures was then added to a PK15 cell monolayer in 96-well culture plates. After 48 h of incubation, the culture plates were fixed for 30 min with absolute ethyl alcohol and subjected to immunofluorescence staining with the monoclonal antibody WH303 (AHVLA, UK; 1:200 diluted in PBS) and FITC-conjugated goat anti-mouse IgG (Santa Cruz Biotech; 1:400 diluted in PBS). The fluorescence signals were observed under a fluorescence microscope (ZEISS), and neutralizing titers were expressed as the reciprocal of the highest dilution that caused complete neutralization.

### Cytokine Measurements

At 0, 7, 14, 21, 28, and 35 dpi, the levels of IFN-γ and IL-4 were determined. Swine PBMCs (1 × 10^6^ cells) were purified from the anterior vena cava of immunized pigs and cultured in 12-well plates. They were treated with inactive purified CSFV (C-strain) for 36 h. IFN-γ and IL-4 in swine PBMC culture supernatants were analyzed using ELISA kits (ExCell Bio, China) according to the manufacturer’s instructions. Standard curves were generated using serially diluted IFN-γ and IL-4 standards. The concentrations of the IFN-γ and IL-4 were calculated according to the corresponding standard curves.

### Quantification of CSFV in Serum Samples

Serum samples were collected from all pigs at 3, 6, 9, and 12 dpc. CSFV genomic copies were detected by real-time PCR. Total RNA in the serum samples was extracted using Transzol UP reagent (Transgen Co., Ltd., Beijing, China). Real-time qRT-PCR amplification was carried out with TransScript Probe one-step qRT-PCR supermix (Vazyme Biotech Co., Ltd.) in a 20-µL reaction mixture containing 10 µL of 2× Supermix, 20 pM of each primer (F: 5′-GCTCCCTGGGTGGTCTAAGTC-3′; R: 5′-GGCTTCTGCTCACGTCGAA-3′), 20 pM of probe (5′-FAM-AGTACAGGACAGTCGTCA-TARAM-3′), 0.5 µL of E-Mix, 0.4 µL of passive reference dye and 4 µL of extracted RNA. The reaction was run using ABI Step One following the manufacturer’s instructions. The reaction was run using the 7300/7500 Real-Time PCR System (Applied Biosystems) with the following program: 15 min at 95°C, followed by 40 cycles of 20 s at 95°C, 30 s at 60°C for 30 s, 1 min at 72°C, and a final extension step of 10 min at 72°C.

### Pathology and Histopathology

The pigs showing signs of irreversible illnesses were humanely euthanized with 100% concentration of CO_2_, and then underwent pathological and histopathological examination following standard operational procedures. All the surviving pigs were humanely euthanized at 15 dpc and underwent pathological and histopathological examination. An extended pathological and histopathological scoring system allowed detailed characterization of pathological lesions (18, 21). Ten parameters (heart, liver, spleen, lung, kidney, stomach, bladder, ileocecal valve, tonsils, and superficial inguinal lymph nodes) were incorporated for the gross pathology and the histopathological scoring systems (Tables 1 and 2). Each parameter was scored from 0 (no lesions) to 3 (severe lesions) (18, 21).

**Table 1:** **Macropathology parameters for postmortem evaluation of pigs infected with highly virulent classical swine fever virus Shimen strain**.

Tissue	Parameters scored
Heart	Hyperemia/petechiae
Liver	Hyperemia/petechiae, necrosis
Spleen	Enlargement/splenomegaly, infarcts
Lung	Hyperemia/petechiae, necrosis
Kidney	Petechiae and discoloration
Stomach	Hyperemia/petechiae, necrosis
Bladder	Hyperemia/petechiae
Ileocecal valve	Ulcer/necrosis
Tonsils	Hyperemia, necrosis
Superficial inguinal lymph nodes	Enlargement and/or hyperemia/petechiae

*Scores applied for each lesion are 0, no lesion; 1, mild; 2, moderate; 3, severe*.

**Table 2:** **Histopathology parameters for evaluation of pigs infected with highly virulent classical swine fever virus Shimen strain**.

Tissue	Parameters scored
Heart	Hyperemia/hemorrhages
Liver	Lymphoplasmacytic infiltrates, hemorrhages
Spleen	Lymphoid depletion, hemorrhages
Lung	Alveolar/septal edema, hemorrhages
Kidney	Hyperemia/hemorrhages, inflammatory infiltrates
Stomach	Hyperemia/hemorrhages
Bladder	Hyperemia/hemorrhages
Ileocecal valve	Necrosis
Tonsils	Lymphoid depletion, hyperemia/hemorrhages
Superficial inguinal lymph nodes	Lymphoid depletion, hyperemia/hemorrhages

*Scores applied for each lesion are 0, no lesion; 1, mild; 2, moderate; 3, severe*.

### Statistical Analysis

The results were represented as mean ± SEM. All data were analyzed using a one-way ANOVA, and values of *P* < 0.05 were considered statistically significant.

## Results

### Construction and Identification of the Recombinant SPV

**Figure 1:**
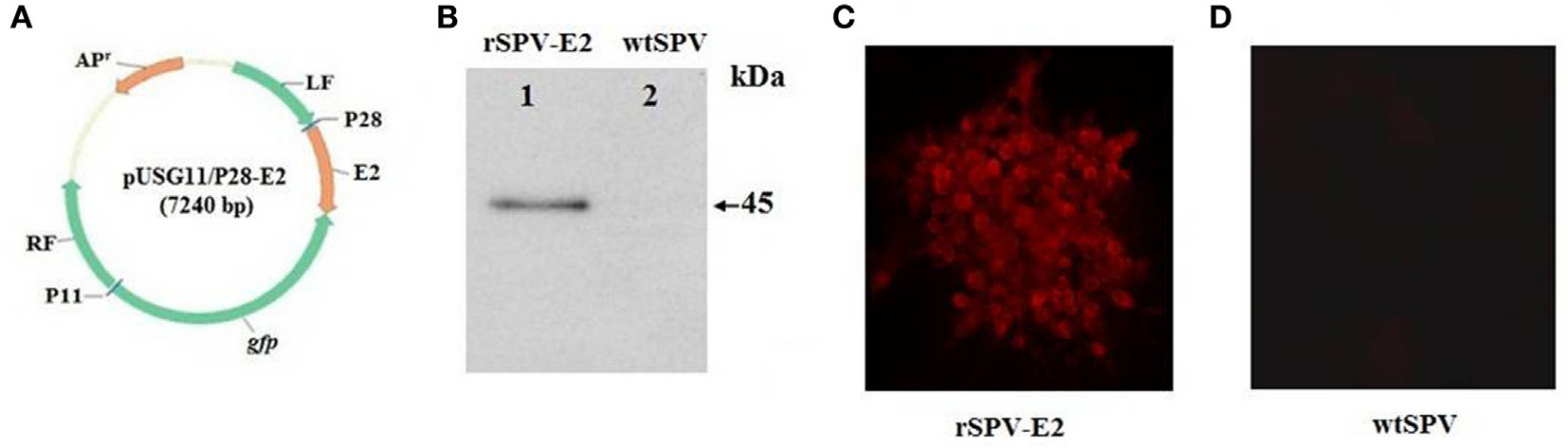
**Construction and identification of the recombinant virus**. **(A)** Construction of the pUSG11/P28-E2 transfer vector. LF and RF indicate the swinepox virus (SPV) left flanking sequences and SPV right flanking sequences, respectively. P11 and P28 are the vaccinia virus promoters. The GFP reporter gene is also included in the plasmid. **(B)** Western blot analysis of E2 expression in PK15 cells: lane 1: PK15 cells infected by rSPV-E2; lane 2: PK15 cells infected by wtSPV. **(C,D)** Indirect immunofluorescence assay of the rSPV-E2. **(C)** Red fluorescence could be observed in rSPV-E2-infected PK15 cells, in which the fluorescence was localized to the cytoplasm. **(D)** No fluorescence was observed in PK15 cells infected with wtSPV.

The 1,218 bp *E2* gene was amplified from the RNA extracted from the CSFV C strain vaccine and inserted into the *Sal*I–*Bam*HI sites of the pUSG11/P28 plasmid to create a pUSG11/P28-E2 plasmid (Figure 1A). The recombinant SPV, rSPV-E2, was then constructed by wtSPV homologous recombination with pUSG11/P28-E2. A Western blot was carried out to verify the expression of E2, approximately 45 kDa in size, in rSPV-E2-infected PK15 cells (Figure 1B). An indirect immunofluorescence assay was used to further verify the expression and localization of E2 in PK15 cells infected with rSPV-E2. A strong red fluorescence signal was observed in the rSPV-E2-infected cells (Figure 1C), whereas no specific red fluorescence was detected in the wtSPV-infected cells (Figure 1D).

### *In Vitro* Determination of Proliferative Capacity and Genetic Stability

Genetic stability of the rSPV-E2 was evaluated in 2 porcine cell lines and 11 non-porcine cell lines. The culture titers of rSPV-E2 remained at about 4.3 × 10^6^ TCID_50_ for more than 60 passages in PK15 and ST porcine cell lines. The rSPV-E2 could not be replicated in Vero, MDBK, or other non-porcine cell lines. After two to three passages, the SPV specific gene of rSPV-E2 could not been detected in the non-porcine cell culture (Table 3). The sequencing results showed that there were no mutations in rSPV-E2 after 60 passages (data not shown), indicating good genetic stability.

**Table 3:** **Titers of rSPV-E2 replication in different cell lines (TCID_50_/log_10_)**.

Passages	1	2	3	4	6	8	10	20	40	60
PK15	6.50	6.47	6.48	6.62	6.57	6.63	6.63	6.59	6.63	6.67
ST	6.52	6.55	6.57	6.52	6.55	6.60	6.62	6.54	6.58	6.63
LLC	2.25	1.20	ND	ND	ND	ND	ND	ND	ND	ND
BHK-21	2.42	1.05	ND	ND	ND	ND	ND	ND	ND	ND
CHO	2.20	1.54	ND	ND	ND	ND	ND	ND	ND	ND
Vero	3.05	2.21	1.00	ND	ND	ND	ND	ND	ND	ND
HEp-2	2.74	ND	ND	ND	ND	ND	ND	ND	ND	ND
HeLa	2.94	1.32	ND	ND	ND	ND	ND	ND	ND	ND
MDCK	1.73	ND	ND	ND	ND	ND	ND	ND	ND	ND
RK13	3.10	1.95	1.15	ND	ND	ND	ND	ND	ND	ND
MDBK	2.22	ND	ND	ND	ND	ND	ND	ND	ND	ND
CEF	2.81	ND	ND	ND	ND	ND	ND	ND	ND	ND
F81	1.55	ND	ND	ND	ND	ND	ND	ND	ND	ND

*ND, not be detected*.

### The Antibody Response to rSPV-E2 Following Vaccination

**Figure 2:**
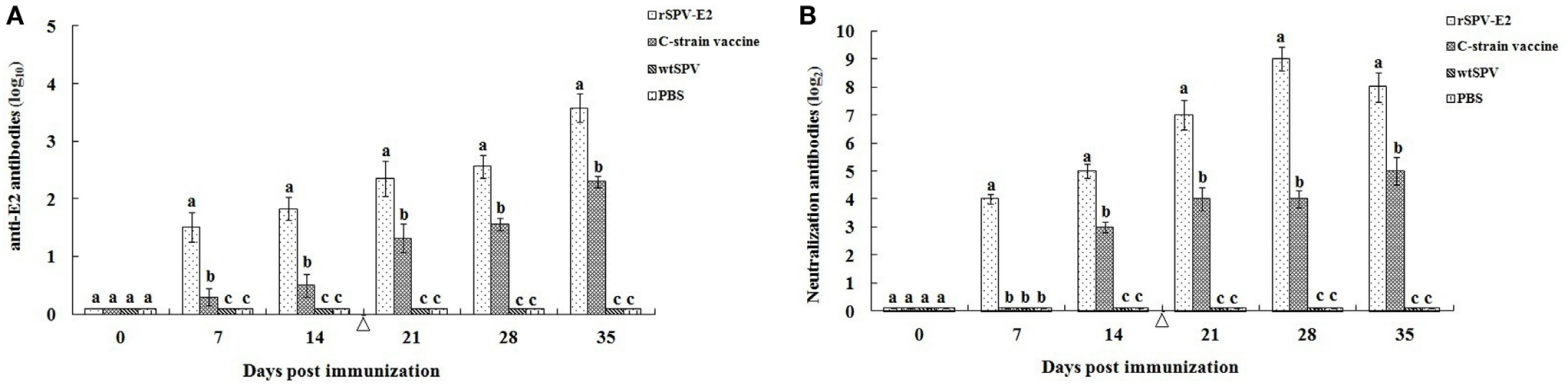
**Antibody response detection in immunized pigs (*n* = 5)**. **(A)** The anti-E2 antibody response to rSPV-E2 following vaccination. **(B)** The classical swine fever virus-specific neutralizing antibody response to rSPV-E2 following vaccination. Different letters (a–c) indicate significant difference (*P* < 0.05) between groups. Δ Indicate the time point (14 dpi) of boost of pigs.

The antibody response elicited after immunization was monitored by determining the serum antibody titers for all the pigs. The anti-E2 antibody titers of the rSPV-E2-immunized group increased following the primary immunization and remained at elevated levels following the second immunization (Figure 2A). The anti-E2 antibody titers of the rSPV-E2-immunized group were significantly higher (*P* < 0.05) than the other three groups (Figure 2A). CSFV-specific neutralizing antibodies in the rSPV-E2-immunized group were elicited at 7 dpi with titers of 1:16, whereas those of the commercial C-strain-vaccine-immunized group were not detected at 7 dpi but appeared at 14 dpi (Figure 2B). At 28 dpi, pigs immunized with rSPV-E2 developed the highest neutralizing antibody concentrations, with titers of 1:512. At all testing time points postimmunization, the CSFV-specific neutralizing antibody titers of the rSPV-E2-immunized group were significantly higher (*P* < 0.05) than that of the commercial C-strain-vaccine-immunized group. In the wtSPV-treated and PBS-treated groups, neither anti-E2 antibodies nor neutralizing antibodies against CSFV could be detected throughout the experiment.

### Detection of the Immune Response

**Figure 3:**
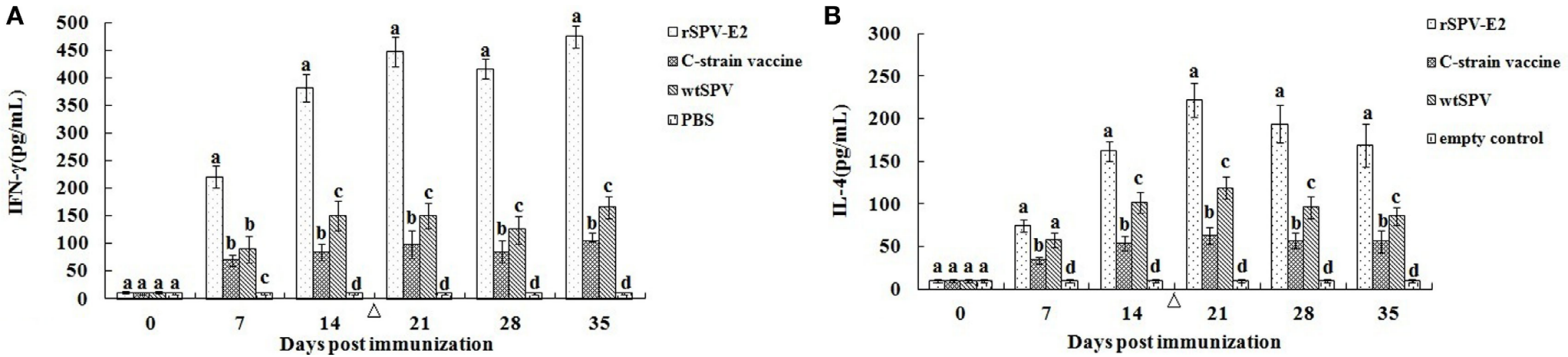
**The immune response to rSPV-E2 following vaccination (*n* = 5)**. The type of immune response induced by rSPV-E2 was indirectly assessed by measuring serum levels of IFN-γ and IL-4 by the ELISA kit. **(A)** The concentration of serum IFN-γ of pigs in different groups postimmunization. **(B)** The concentration of serum IL-4 of pigs in different groups postimmunization. Different letters (a–d) indicate significant difference (*P* < 0.05) between groups. Δ Indicate the time point (14 dpi) of boost of pigs.

The immune response of the body is mainly induced by Th1 and Th2 T cell subsets. Th1 cells mainly produce IFN-γ, IL-2, and TNF-β and primarily induce cellular immune and inflammatory responses; Th2 cells mainly produce IL-4, IL-6, and IL-10, and mainly induced humoral immune response and eosinophil accumulation. The cellular and humoral immune response induced by rSPV-E2 was indirectly assessed by measuring serum levels of IFN-γ and IL-4 by the ELISA kit. As shown in Figure 3, the rSPV-E2 immunized group induced a significantly higher level of IFN-γ and IL-4 compared to the other three groups (*P* < 0.05). This indicated that both the Th1-type and Th2-type immune responses were enhanced in rSPV-E2-immunized animals. Interestingly, the serum levels of IFN-γ and IL-4 of the wtSPV immunized group were significantly higher than the PBS-treated group (*P* < 0.05), which indicated that the SPV vector could be used as an adjuvant to enhance the immune response.

### Quantification of CSFV in the Serum Samples

**Figure 4:**
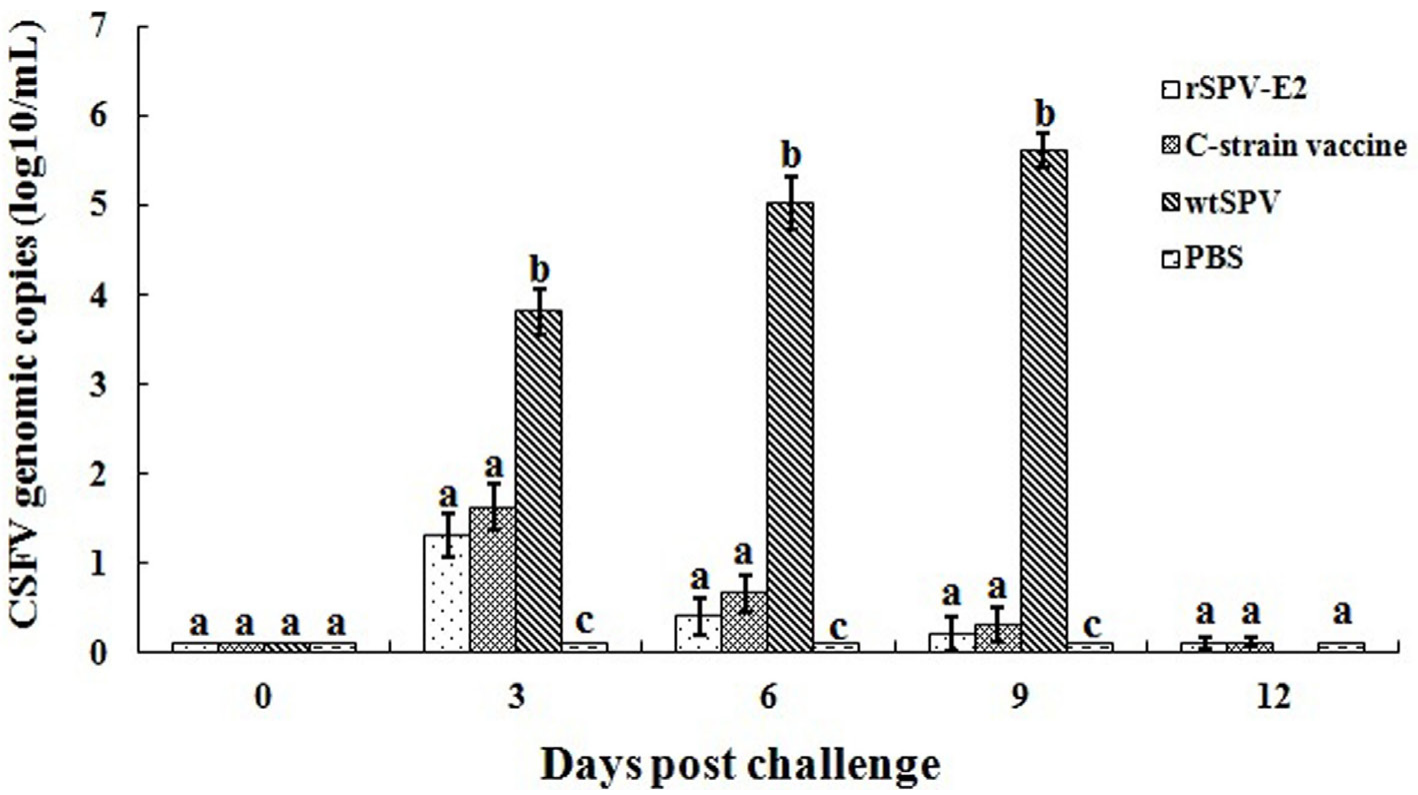
**Quantification of classical swine fever virus (CSFV) in the serum samples following vaccination (*n* = 5)**. Serum samples were collected from all pigs at 3, 6, 9, and 12 dpc. CSFV genomic copies were detected by real-time PCR. Different letters (a–c) indicate significant difference (*P* < 0.05) between groups.

Serum samples were collected from all pigs at 3, 6, 9, and 12 dpc. CSFV genomic copies were detected by real-time PCR. CSFV genomic copies were not detected in any of the serum samples at 0 days post-challenge (dpc) in any of the pigs. The copies were significantly increased by 3 dpc. The serum CSFV genomic copies in the rSPV-E2-immunized group and the commercial C-strain-vaccine-immunized group were significantly lower (*P* < 0.01) compared with the wtSPV-treated group for all time points post-challenge (Figure 4).

### Clinical Evaluation

**Figure 5:**
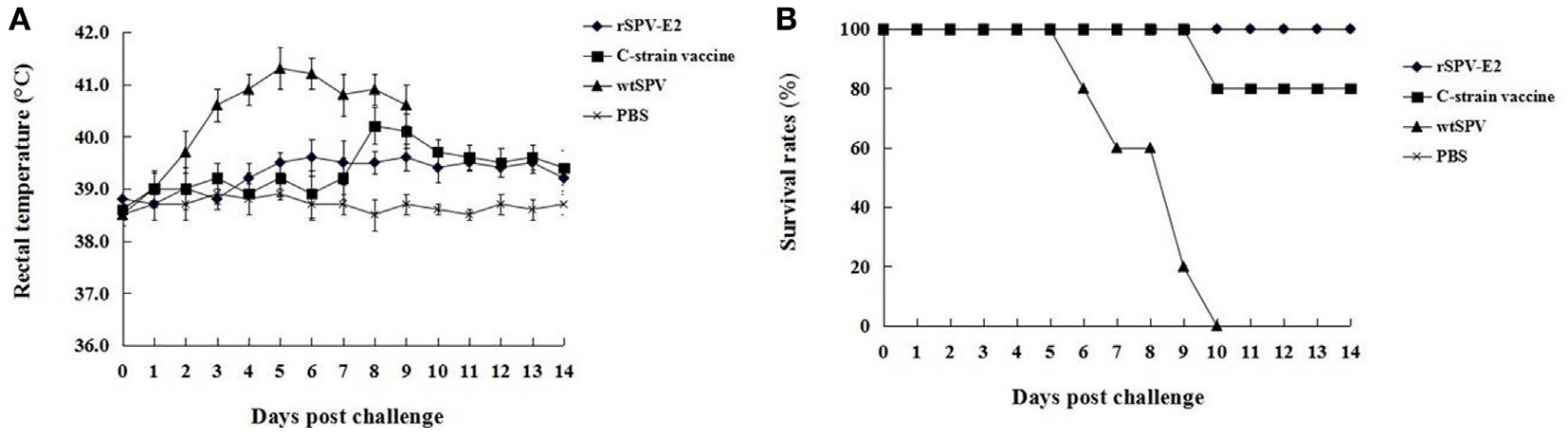
**Clinical evaluation of pigs in different groups (*n* = 5) post-classical swine fever virus (CSFV) challenge**. **(A)** Mean rectal temperatures of pigs in different groups post-CSFV challenge. **(B)** The survival rates of pigs in different groups post-CSFV challenge.

The clinical symptoms and survival rates of the pigs were monitored. Prior to CSFV challenge, no clinical signs were observed in any of the four groups. Post-CSFV challenge, all pigs in the wtSPV-treated group developed typical CSF signs from 3 dpc, such as pyrexia (>40°C), weakness, drowsiness, huddling, anorexia, an unsteady gait, and convulsions. The mean rectal temperatures of the pigs in the wtSPV-treated group were significantly higher (*P* < 0.05) than in the rSPV-E2-immunized group and commercial C-strain vaccine immunized group from 3 to 9 dpc (Figure 5A). All pigs in the wtSPV-treated group which showing signs of irreversible illnesses were humanely euthanized with 100% concentration of CO_2_ (Figure 5B). One pig in the commercial C-strain vaccine immunized group showed detectable CSF signs at 8 dpc and showed signs of irreversible illnesses at 10 dpc, and then was humanely euthanized with 100% concentration of CO_2_. No pigs in the rSPV-E2 immunized group showed detectable CSF signs or died during the experiment.

### Pathology and Histopathology

Pigs that showing signs of irreversible illnesses were humanely euthanized with 100% concentration of CO_2_, and then underwent pathological and histopathological examination following standard operational procedures. All the surviving pigs were humanely euthanized at 15 dpc and underwent pathological and histopathological examination. Pigs in the wtSPV-treated group showed lesions typical of CSF after challenge, such as pinpoint hemorrhage in the kidney or hemorrhage within the lymph nodes and bladder, necrosis in the tonsils, hydropericardium, lymphadenectasis, splenic infarcts, and petechiae. There were also obvious lesions in the liver and lungs, and the stomach showed large areas of hemorrhage and ulcers. The scores for the pathological changes in each group are shown in Table 4.

**Table 4:** **Average scores for the pathological changes of challenged pigs**.

Groups	Heart	Liver	Spleen	Lung	Kidney	Stomach	Bladder	Ileocecal valve	Tonsils	Superficial inguinal lymph nodes	Total
rSPV-E2	0.2	0.4	0.4	0.6	0.8	0.4	0.6	0.6	0.2	0.4	4.6
C-strain vaccine	0.2	0.6	0.8	0.8	1.0	0.4	0.8	0.6	0.6	0.4	6.2
wtSPV	1.2	1.8	2.6	2.2	2.8	1.6	2.2	1.6	2.2	1.8	20.0
PBS (NC)	0	0	0	0	0	0	0	0	0	0	0

**Figure 6:**
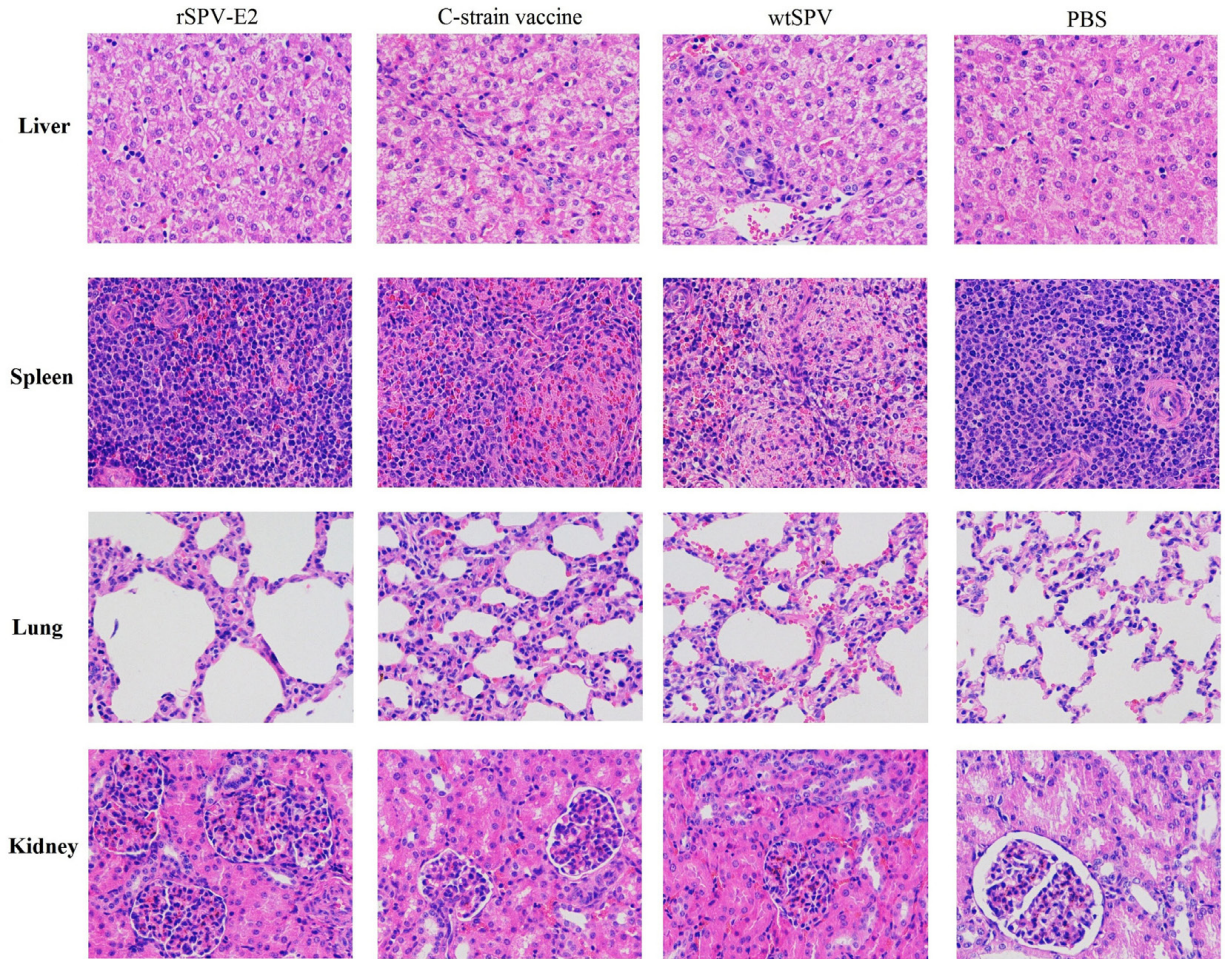
**The histopathological changes (200×) of liver, spleen, lung, and kidney of pigs in different groups**.

Infected pigs also showed severe hemorrhaging within the stomach, intestinal tract and mesentery. The most common histopathological findings in the wtSPV-treated group were mild to severe lymphoid depletion, accompanied by hyperemia and hemorrhage. Differing degrees of lymphoid depletion were also observed in the spleen. Perivascular cuffing due to inflammatory lymphohistiocytic infiltrates was frequently observed in the liver and kidney (Figure 6). Pigs in the wtSPV-treated group (no-challenge control) showed no obvious changes. The scores for the histopathological changes in each group are shown in Table 5. These data indicate that the histopathological changes seen in the pigs in the wtSPV-treated group were more severe than those in the rSPV-E2-immunized group and the commercial C-strain-vaccine-immunized group. No statistically significant differences were observed between the rSPV-E2-immunized group and the commercial C-strain-vaccine-immunized group.

**Table 5:** **Average scores for the histopathological changes of challenged pigs**.

Groups	Heart	Liver	Spleen	Lung	Kidney	Stomach	Bladder	Ileocecal valve	Tonsils	Superficial inguinal lymph nodes	Total
rSPV-E2	0	0.2	0.2	0.4	0.6	0.2	0.4	0.4	0.2	0.4	3.0
C-strain vaccine	0	0.4	0.6	0.4	0.8	0.4	0.6	0.4	0.4	0.4	4.4
wtSPV	0.8	1.4	2.2	1.8	2.4	1.2	1.8	1.2	1.4	1.2	15.4
PBS (NC)	0	0	0	0	0	0	0	0	0	0	0

## Discussion

Classical swine fever virus infection in swine results in a highly contagious and severe disease that is characterized by fever and hemorrhage (22, 23). The attenuated vaccines against CSF, such as the C-strain, protects pigs from clinical CSF disease, however, do not provide for serological discrimination between postinfectious and vaccine-induced immunity. This aspect is especially significant in detecting chronically infected animals, which do not develop any clinical signs of the disease for a long time, and thus may be a source of spreading virulent CSFV in the livestock. The recombinant SPV expressing glycoprotein E2 of CSFV enables DIVA and is benefit for CSF control and eradication program, particularly in the later stages of an eradication campaign and for countries where the disease is not endemic.

Classical swine fever virus-neutralizing antibodies play a critical role in the clearance of the virus and can protect pigs against CSFV infection (24). An ideal CSFV vaccine should be able to induce a rapid and robust neutralizing antibody response following vaccination. Madera et al. constructed an E2 subunit vaccine KNB-E2, which was formulated with the recombinant E2 protein (genotype 1.1) expressed by insect cells and an oil-in-water emulsion based adjuvant (25). The KNB-E2 vaccine could develop high levels of E2-specific antibodies and anti-CSFV-neutralizing antibodies and reduce the level of CSFV load in blood and nasal fluid post-CSFV challenge. Xia et al. found that the recombinant adenovirus expressing CSFV E2 (rAdV-SFV-E2) could induce complete protection to piglets against the challenge of highly virulent CSFV Shimen strain (26). Tian et al. constructed a multiple-epitope recombinant vaccine, which was composed of two copies each of glycoprotein E2 residues 693–707, 241–276, and 770–781, and two copies amino acid residues 1446–1460 of the non-structural protein NS2-3. This multiple-epitope recombinant vaccine could stimulate pigs to produce protective neutralization antibodies and delay the clinical development time from CSFV challenge (27). In this study, the glycoprotein E2 of CSFV was expressed using the swinepox expression system, and its immunogenicity was tested in pigs. At 7 dpi, pigs in the rSPV-E2-immunized group produced CSFV-specific neutralizing antibodies with titers of 1:16, whereas no CSFV-specific neutralizing antibodies were detected in the commercial C-strain-vaccine-immunized group until 14 dpi. At 28 dpi, pigs immunized with rSPV-E2 developed the highest CSFV-specific neutralizing antibody concentrations with titers of 1:512. At all testing time points post-immunity, the CSFV-specific neutralizing antibody titers of the rSPV-E2-immunized group were significantly higher than the commercial C-strain-vaccine-immunized group (*P* < 0.05). At 35 dpi, groups 1, 2, and 3 were intranasally challenged with 1 × 10^5^ TCID_50_ CSFV Shimen strain per piglet. One pig in the commercial C-strain-vaccine-immunized group showed signs of irreversible illnesses and then was humanely euthanized with 100% concentration of CO_2_, it might due to the low CSFV-specific neutralizing antibody titer of this pig (1:8), while the CSFV-specific neutralizing antibody titers of the other pigs in this group were 1:64. The results showed that rSPV-E2 induced a substantial CSFV-specific neutralizing antibody response in immunized pigs and could provide pigs with significant protection from CSFV infection.

The complexity of the immune response to CSFV and the ability of the virus to escape or modulate the host immune system make it difficult to develop a vaccine that can be used to eradicate the disease (28, 29). SPVs are promising candidates for vaccine vectors due to the ability to induce of both cellular and humoral immunity, and the capacity for heterogeneous insertions (20). The natural infection rate of SPV in China is less than 5%. Van der Leek et al. constructed a recombinant swinepox virus-Aujeszky’s disease (rSPV-AD) that could induce serum-neutralizing antibodies to Aujeszky’s disease virus in pigs. After vaccination with rSPV-AD, the serum-neutralizing antibodies to Aujeszky’s disease virus persisted for 150 days, and all the pigs showed an anamnestic response when they were revaccinated. In the present study, we constructed a recombinant SPV, rSPV-E2 that expressed the glycoprotein E2 for CSFV. The results of the animal experiment demonstrate that rSPV-E2 is capable of inducing both the humoral immune response and the cellular immune response and could protect the challenged pigs from viremia. Therefore, it may serve as a promising candidate vaccine against CSFV infection. There are often multiple infections in most pig farms in China, such as CSFV, porcine reproductive and respiratory syndrome virus, porcine circovirus type 2, and porcine epidemic diarrhea virus, so in future work, we will investigate the construction of a recombinant SPV expressing two or more protective antigens of swine pathogens.

## Ethics Statement

All experimental protocols were approved by the Laboratory Animal Monitoring Committee of Jiangsu province and performed accordingly (Government Degree No. SYXK2015-0036).

## Author Contributions

HL and HF designed the study. LC performed the animal experiment. ZM performed the date analysis. All the authors read and approved the final manuscript.

## Conflict of Interest Statement

The authors declare that the research was conducted in the absence of any commercial or financial relationships that could be construed as a potential conflict of interest.
